# Incremental Structured Dictionary Learning for Video Sensor-Based Object Tracking

**DOI:** 10.3390/s140203130

**Published:** 2014-02-17

**Authors:** Ming Xue, Hua Yang, Shibao Zheng, Yi Zhou, Zhenghua Yu

**Affiliations:** 1 Institute of Image Communication and Network Engineering, Shanghai Jiao Tong University, Shanghai 200240, China; E-Mails: hyang@sjtu.edu.cn (H.Y.); sbzh@sjtu.edu.cn (S.Z.); 2 Shanghai Key Laboratory of Digital Media Processing and Transmissions, Shanghai 200240, China; 3 Department of Electronics Engineering, Dalian Maritime University, Dalian 116026, China; E-Mail: zhouyi21st@gmail.com; 4 Bocom Smart Network Technologies Inc., Shanghai 200233, China; E-Mail: zhenghua.jack.yu@gmail.com

**Keywords:** appearance model, object tracking, sparse representation, structured dictionary learning, Bayesian inference, visual sensor networks

## Abstract

To tackle robust object tracking for video sensor-based applications, an online discriminative algorithm based on incremental discriminative structured dictionary learning (IDSDL-VT) is presented. In our framework, a discriminative dictionary combining both positive, negative and trivial patches is designed to sparsely represent the overlapped target patches. Then, a local update (LU) strategy is proposed for sparse coefficient learning. To formulate the training and classification process, a multiple linear classifier group based on a K-combined voting (KCV) function is proposed. As the dictionary evolves, the models are also trained to timely adapt the target appearance variation. Qualitative and quantitative evaluations on challenging image sequences compared with state-of-the-art algorithms demonstrate that the proposed tracking algorithm achieves a more favorable performance. We also illustrate its relay application in visual sensor networks.

## 1. Introduction

Object tracking via video sensors is an important subject and has long been investigated in the computer vision community. In common sense, an object, or a target, refers to a region in the video frame detected or labeled for specific purposes. Stable and accurate tracking of objects is fundamental to many real-world applications, such as motion-based recognition, automated surveillance, visual sensor network, video indexing, human-computer interaction, traffic monitoring, vehicle navigation, *etc.* [1].

Historically, visual trackers proposed in the early years typically kept the appearance model fixed throughout an image sequence. Recently, methods proposed to track targets while evolving the appearance model in an online manner, called online visual tracking, have been popular [2]. An online visual tracking method typically follows the Bayesian inference framework and mainly consists of three components: an object representation scheme, which considers the appearance formulation uniqueness of the target; a dynamical model (or state transition model), which aims to describe the states of the target and their inter-frame relationship over time; an observation model, which evaluates the likelihood of an observed image candidate (associated with a state) belonging to the object class. Although visual tracking has been intensively investigated, there are still many challenges, such as occlusions, appearance changes, significant motions, background clutter, *etc*. These challenges make the establishment of an efficient online visual tracker a difficult task.

### 1.1. Related Works

Appearance representation of the target is a basic, but important, task for visual tracking. Discrimination capability, computational efficiency and occlusion resistance are generally considered as the three main aspects in appearance modeling. For online visual tracking, the schemes can be classified into patch-based schemes (e.g., holistic gray-level image vector [3] and fragments [4–6]), feature-based schemes [7–10], statistics-based schemes [11–15] and their combinations.

Based on the differences in object observation modeling, online visual tracking can be generally classified into generative methods (e.g., [3,4,11–13,15–18]), discriminative methods (e.g., [7–10,15]) and hybrid methods (e.g., [19,20]). Generative methods focus on the exploration of a target observation with minimal predefined error based on separative evaluation criteria, while discriminative ones make attempts to maximize the margin or inter-class separability between the target and non-target regions using classification techniques. Typical techniques include boosting [8,9] and support vector machine (SVM) [7,21,22]. For the trackers using SVM, Avidan *et al.* [7] propose a tracking algorithm integrating SVM to discriminate the target from its background. Tian *et al.* [21] present a tracking system based on an ensemble of adaptively-weighted linear SVM classifiers based on their discriminative abilities. Bai and Tang *et al.* [22] propose an online Laplacian ranking support vector tracker (LRSVT), which incorporates the weakly labeled information to resist full occlusion and adapt to target appearance variation. Yet, there are still some limitations for these works. Firstly, most of them consider the classification problem on a single-patch level, which might lack flexibility and robustness when a drastic appearance occurs. Secondly, the features applied in these works are not unique enough. It could negatively influence the tracking performance when a similar object exists. In this paper, we continue to explore the application of SVM classifiers in online visual tracking, where the input features are coefficients of sparse representation on a patch level. Thus, the patch-based SVMs are grouped for classifier modeling.

As an elegant working model, sparse representation has recently been extensively studied and applied in pattern recognition and computer vision [23,24]. There are two basic problems [25]: the first one is to calculate the sparse solution of a linear system, while the second one refers to learning a suitable dictionary for approximation performance improvement. So far, the former one has been deeply exploring in visual tracking (e.g., [11–13,15,17,20]). Within the particle filtering framework, most of the works cast the tracking problem as searching the most likely sampling candidate of the target via *l*_1_ minimization. Mei and Ling [11] apply sparse representation to visual tracking and deal with occlusions via positive and negative trivial templates. Wang *et al.* [17] propose a novel online object tracking algorithm with sparse prototypes, which adopts principal component analysis (PCA) basis vectors and trivial templates to represent the tracked target sparsely, and solve the problem by using an iterative thresholding method. Zhong *et al.* [20] develop a hybrid tracking method, where a sparsity-based discriminative classifier (SDC) and a sparsity-based generative model (SGM) are cascaded for target location estimation. However, investigation of the second problem in visual tracking has just started. Liu *et al.* [12] develop a generative visual tracking algorithm with a static sparse dictionary of the target and dynamically online updated basis distribution model by K-selection, while a recent method proposed by Wang *et al.* [15] discriminates the target from the background based on the classification of the sparse coefficients with an over-complete dictionary without learning. Learning a dictionary for classification has recently been popular [26–28], which adds specific constraints in the dictionary learning (DL) model to gain discrimination ability. In these works, constraints have been considered on the class labels, learning process and sparse representation coefficients, and discriminability has been enforced in sparse codes during the dictionary learning process to improve classification accuracy. However, they fail to consider the dictionary design from a discriminative perspective. Moreover, the dictionary is often learned as a whole, and the learning process is very time consuming, which might not be suitable for a recognition application in continuous appearance variation circumstances.

### 1.2. Our Proposal

**Figure 1.**
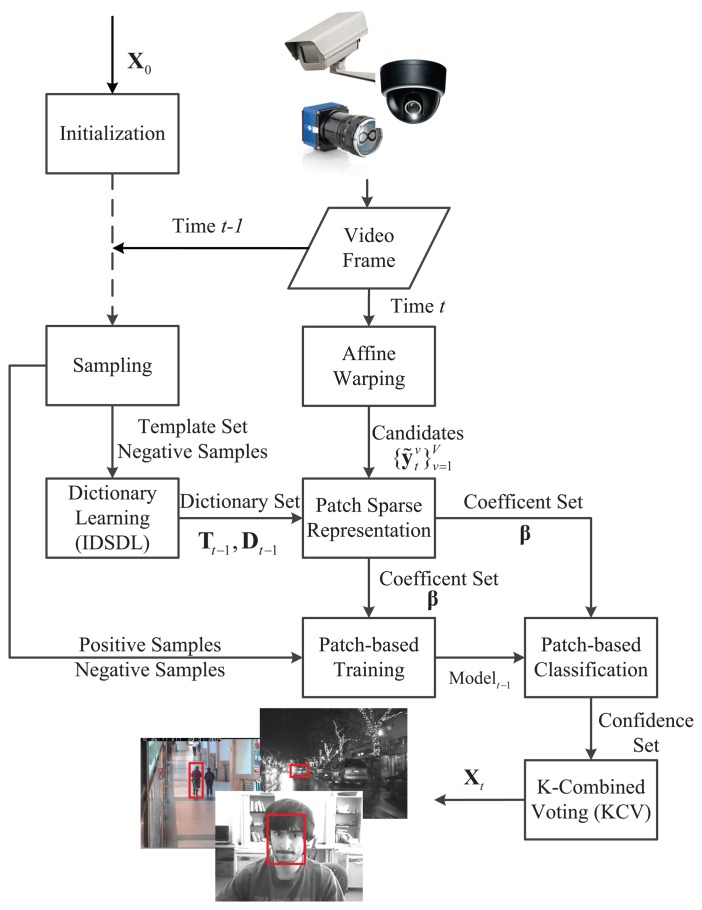
Workflow of the proposed algorithm. The proposed dictionary learning method, incremental discriminative structured dictionary learning (IDSDL), is detailed in Section 2, while the proposed affine warping, support vector machine (SVM) training and classification and K-combined voting (KCV) are detailed in Section 3.

Inspired by the discussions above, this paper considers the dictionary learning problem for online visual tracking, as well as a visual tracking algorithm, incremental discriminative structured dictionary learning (IDSDL)-VT, including incremental discriminative structured dictionary learning and multiple linear classifiers. The workflow is shown in Figure 1. On a patch level, groups of positive and negative sparse coefficients of the target patches learned by the proposed incremental discriminative structured dictionary learning (IDSDL) algorithm are input to the support vector machines (SVMs) to train classifiers, which discriminate the target from the background. In the next frame, target candidates are sampled based on affine warping, and their corresponding coefficients are obtained. Then, given the learned model set, patch-based classifications are conducted to calculate the confidence set, and a K-combined voting (KCV) function is used to jointly locate the target. The dictionary set is incrementally adapted as time evolves.

Compared with the dictionary learning papers referred to above, we do not solely rely on the optimization, but focus on the dictionary design with separate learning to improve the discriminative ability of the sparse coefficients for classification. Moreover, the dictionary is built on a patch level, and thus, a spatial multi-dictionary learning structure is established. Though numbers of papers have appeared based on sparse representation, few consider the dictionary learning aspect. There is a dictionary learning process in the algorithm proposed by Liu *et al.* [12], but it is a generative approach. Moreover, the algorithm proposed by Zhong *et al.* [20] is a hybrid one, and its discriminative ability is not based on the binary classifier, but the reconstruction error, which is used to generatively create weights for confidence modeling. The algorithm proposed by Wang *et al.* [15] is discriminative, yet constructed without learning. Differently, in this paper, we exploit dictionary learning within the discriminative tracking framework and establish a tracking process based on patch-based classifiers. The main contributions of the proposed algorithm can be described as follows: (1) compared with the previous sparse-representation-based tracking algorithms referred to above, a structured dictionary learning algorithm for discriminative classification is newly proposed; (2) compared with the generative tracking framework via the dictionary learning summarized above, the proposed approach integrates the learning process to formulate a discriminative visual tracking framework, which learns multiple classifiers on a structured level; (3) compared with a one-time peak-confidence calculation, a K-combined voting (KCV) function based on multiple classifier confidences is novelly proposed to locate the target. We focus on the dictionary design part rather than the optimization process. Experiments on both a single camera and visual sensor network are conducted to demonstrate the performance of the proposed method.

The rest of the paper is organized as follows. In Section 2, details of the proposed structured dictionary learning algorithm are described. Details of the proposed visual tracking algorithm within the Bayesian inference framework are proposed in Section 3. Experimental results and a discussion are given in Section 4. In Section 5, concluding remarks and a possible direction for future research are provided.

## 2. Discriminative Structured Dictionary Learning

We begin the description of the proposed dictionary learning algorithm, incremental discriminative structured dictionary learning (IDSDL), with the sparse appearance modeling as follows. Typically, the global appearance of an object under different illumination and viewpoint conditions is known to lie approximately in a low-dimensional subspace [11]. Basically, we assume that the target could be represented with a lower error by its overlapped patches in the form of the target templates' learning results in the previous frames. The template contains a set of images, each of which is cropped from the corresponding video frame based on the latest tracking results. Similar assumptions are also applied in other tracking algorithms based on sparse representation [11-13,15,17,20].

Suppose at time *t*, the target ${\text{Y}}_{t}=\left[{\text{p}}_{t}^{1},{\text{p}}_{t}^{2},\ldots ,{\text{p}}_{t}^{N}\right]\in {\mathbb{R}}^{d^{2}\times N}$ is sampled and vectorized into *N* separate overlapped patches with zero mean and unit variance, where the size of each patch is *d*^2^ × 1. Moreover, there exists a set of templates ${\text{T}}_{t}=\left[{\text{t}}_{t}^{1},{\text{t}}_{t}^{2},\ldots ,{\text{t}}_{t}^{M}\right]\in {\mathbb{R}}^{d^{2}\times N\times M}$, where *M* refers to the number of the templates, and the corresponding patches ${\text{t}}_{t}^{j}={\left[{\text{b}}_{j}^{1},{\text{b}}_{j}^{2},\ldots ,{\text{b}}_{j}^{N}\right]}^{T}\in {\mathbb{R}}^{d^{2}\times N}$, *j* = 1,2,…, *M* share the same patch sampling scheme with that of the target candidates and have been stacked, normalized and vectorized. Therefore, in the current frame, any patch of the target candidate ${\text{p}}_{t}^{i}\in {\mathbb{R}}^{d^{2}}$, *i* = 1, 2, …, *N* could approximately lie in the linear span of the corresponding template patches:

(1) $${\text{P}}_{t}^{i}=\beta_{1}^{i}{\text{b}}_{t}^{1}+\beta_{2}^{i}b_{t}^{2}+\cdots \beta_{M}^{i}b_{t}^{M}$$

for some scalars, $\beta_{j}^{i}\in \mathbb{R}$, *j* = 1, 2, …, *M*. Thus, $p_{t}^{i}$ could be represented based on the templates by solving the optimization problem based on the elastic net regularization [29] using the least angle regression (LARS) method:

(2) $$\begin{array}{l} \underset{\beta^{i}\in {\mathbb{R}}^{M}}{\min}\frac{1}{2}{\left\|{\text{p}}_{t}^{i}-{\text{T}}_{t}^{i}\beta^{i}\right\|}_{2}^{2}+\lambda_{1}{\left\|\beta^{i}\right\|}_{1}+\frac{\lambda_{2}}{2}{\left\|\beta^{i}\right\|}_{2}^{2}.\\ \text{s}.\text{t}.\beta^{i}\geq 0 \end{array}$$

where *λ*_1_ and *λ*_2_ are regularization constants. Most of the previous tracking works apply the *l*_1_ constraints to the coefficients, which is an approximation to the *l*_0_ regularizer for the purpose of convexity, and the sparsity-based optimization methods by previous tracking works include basis pursuit (BP) [11] and orthogonal matching pursuit (OMP) [13]. In this paper, we apply the elastic net penalty [29], which is a convex combination of the lasso and ridge penalty, and are able to conduct automatic variable selection and continuous shrinkage, select groups of correlated variables and try to avoid overshrink in regression problems.

In sparse representation, a dictionary refers to a matrix **D** = [**d**_1_, **d**_2_,…,**d**_*n*_] ∈ ℝ^(*d*^2^×*N*)×*M*^ made up of a group of basis vectors, where the target signal is spanned. Given a training set of image patches, **Y**, classical dictionary learning methods learn an optimized dictionary, **D**, by solving the following objective function:

(3) $$\begin{array}{l} \underset{\text{D},\beta }{\min}\sum_{i=1}^{N}\left[\frac{1}{2}{\left\|p_{t}^{i}-\text{D}\beta_{i}\right\|}_{2}^{2}+\lambda {\left\|\beta_{i}\right\|}_{1}\right]\\ \text{s}.\text{t.}{\left\|d_{j}\right\|}_{2}^{2}\leq 1,\forall j, \end{array}$$

This problem is not jointly convex with respect to **D** and *β*, and it is commonly solved by alternating between the two variables. Recent studies have shown surprisingly promising results in image classification, when the dictionary size is sufficiently large. However, for the application of visual tracking, the size cannot be very high for computational efficiency. On the other hand, a fixed dictionary is generally not sufficient to cope with the appearance changes of the tracking object, as well as the background.

**Figure 2.**
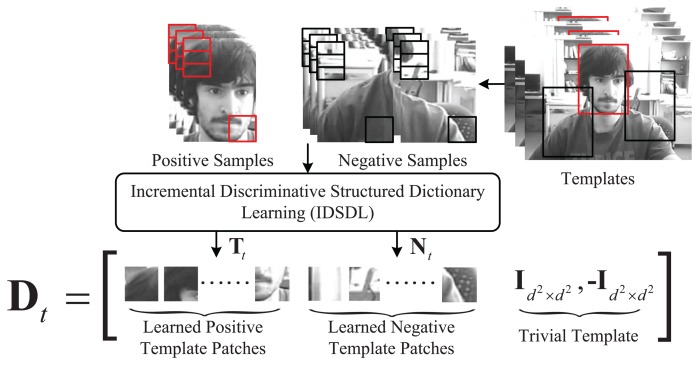
Generation of proposed dictionary, which is composed of positive and negative template patches learned by IDSDL and trivial templates. The corresponding patches are cropped separately from the positive and negative samples around the target based on different sampling radii.

**Figure 3.**
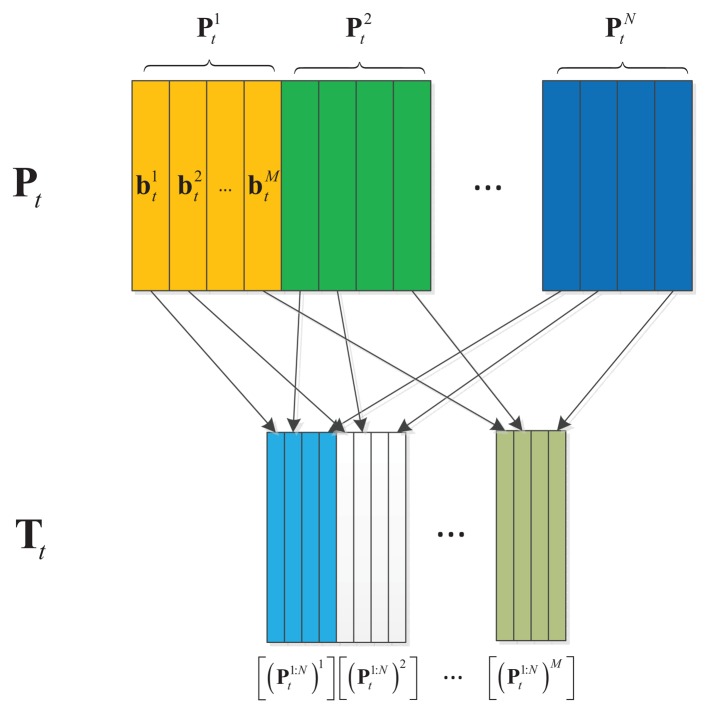
Relationship between ${\text{p}}_{t}^{i}$ and T*_t_*, where $\left[{\left({\text{p}}_{t}^{1:N}\right)}^{i}\right]\in {\mathbb{R}}^{d^{2}\times M}$ refers to the matrix composed of *M* columns separately containing the *i*-th column in non-vectorized matrix ${\text{t}}_{t}^{j}\in {\mathbb{R}}^{d^{2}\times N}$, *j* = 1,2,…, *M*.

Based on the assumption and definition described above, we present an incremental discriminative structured dictionary learning method. A structured dictionary is defined as ${\text{D}}_{t}={\left\{{\text{D}}_{t}^{i}\right\}}_{i=1}^{N}$, as shown in Figure 2, where ${\text{D}}_{t}^{i}$ is its element corresponding to the *i*-th patch. Furthermore, ${\text{D}}_{t}^{i}$ is defined to be constructed as:

(4) $${\text{D}}_{t}^{i}=\left[{\text{T}}_{t},{\text{N}}_{t}^{i},\text{I},-\text{I}\right]$$

(5) $${\text{T}}_{t}=\left[\left[{\left({\text{P}}_{t}^{1:N}\right)}^{1}\right],\left[{\left({\text{P}}_{t}^{1:N}\right)}^{2}\right],\ldots ,\left[{\left({\text{P}}_{t}^{1:N}\right)}^{M}\right]\right]$$

(6) $${\text{P}}_{t}^{i}=\left[{\text{b}}_{t}^{1},{\text{b}}_{t}^{2},\ldots ,{\text{b}}_{t}^{M}\right],{\text{N}}_{t}^{i}=\left[{\text{d}}_{t}^{1},{\text{d}}_{t}^{2},\ldots ,{\text{d}}_{t}^{M}\right]$$

where $\left[{\left({\text{P}}_{t}^{1:N}\right)}^{j}\right]\in {\mathbb{R}}^{d^{2}\times N}$ refers to the matrix composed of *N* columns separately containing the *j*-th column of a matrix ${\text{P}}_{t}^{i}\in {\mathbb{R}}^{d^{2}\times M}$, *i* = 1, 2, …, *N*. ${\text{P}}_{t}^{i}\in {\mathbb{R}}^{d^{2}\times M}$ refers to the dictionary part learned by ${\text{p}}_{s^{+}}^{i}$, and ${\text{N}}_{t}^{i}\in {\mathbb{R}}^{d^{2}\times M}$ refers to the part by ${\text{p}}_{s^{-}}^{i}$, where ${\text{b}}_{t}^{j}$ and ${\text{d}}_{t}^{j}$ share the same patch sampling scheme with that of the target candidate. The relationship between ${\text{P}}_{t}^{i}$ and T*_t_* is shown in Figure 3. **I** ∈ ℝ^*d*^2^×*d*^2^^ is an identity matrix used as a non-negativity constraints similar with the settings in [11,15].

Suppose the target location has been estimated; the positive and negative training samples could be represented in the overlapped form by ${\text{S}}_{t}={\left\{{\text{p}}_{t}^{i},l_{i}\right\}}_{i=1}^{N}$, *l_i_* ∈ {+1, −1}. Corresponding patches are ${\text{p}}_{t}^{i}={\left\{x_{k}^{i},l_{k}^{i}\right\}}_{k=1}^{s}$ and *s* = *s*^+^ + *s*^−^, which separately refer to the number of positive samples, ${\text{p}}_{s^{+}}^{i}$, and negative ones, ${\text{p}}_{s^{-}}^{i}$. A local update (LU) strategy is introduced to both update the dictionary and improve the inter-patch independence and separability. For each patch in ${\text{p}}_{s^{+}}^{i}$ only the corresponding *M* columns of the dictionary $\left[{\left({\text{P}}_{t}^{1:N}\right)}^{i}\right]$ with the same patch index are learned and temporarily stacked, while the rest columns stay fixed. **T***_t_* is not replaced until each column is updated. On the other hand, after ${\text{p}}_{s^{-}}^{i}$ is sparsely represented, ${\text{N}}_{t}^{i}$ is directly updated. The update process [30] is defined as:

(7) $${\text{u}}_{j}=\frac{1}{{\text{A}}^{jj}}\left({\left({\text{B}}_{t}\right)}^{\left(j\right)}-{\text{D}}_{t-1}^{i}{\left({\text{A}}_{t}\right)}^{\left(j\right)}\right)+{\left({\text{D}}_{t-1}^{i}\right)}^{\left(j\right)}$$

(8) $${\left({\text{D}}_{t}^{i}\right)}^{\left(j\right)}=\frac{1}{\max\left({\left\|{\text{u}}_{j}\right\|}_{2},1\right)+\delta }{\text{u}}_{j}$$

where *δ* is a small constant, and (·)^(^*^j^*^)^ refers to the *j*-th column. ${\text{A}}_{t}={\sum_{t=0}^{t}\beta_{t}^{i}\left(\beta_{t}^{i}\right)}^{T}\in {\mathbb{R}}^{M\times M}$ in and ${\text{B}}_{t}={\sum_{t=t_{0}}^{t}{\text{p}}_{t}^{i}\left(\beta_{t}^{i}\right)}^{T}\in {\mathbb{R}}^{d^{2}\times M}$ are two auxiliary matrices. Equations (7) and (8) sequentially update each column of the dictionary, while keeping the other ones fixed under a potential constraint ${\left({\left({\text{D}}_{t}^{i}\right)}^{\left(j\right)}\right)}^{T}{\left({\text{D}}_{t}^{i}\right)}^{\left(j\right)}\leq 1$. This results in an orthogonal projection of the vector, **u**_*j*_, onto the constraint set, namely the *l*_2_ – *ball* in this paper. Convergence to a global optimum is guaranteed, since the convex optimization problem admits separable constraints in the updated columns. As time evolves, the value of **D***_t_*_−1_ is a warm start for **D***_t_*, and a single iteration has been empirically found to be sufficient for the convergence of the dictionary update step [30]. The proposed algorithm is listed in Algorithm 1.



**Algorithm 1** Incremental discriminative structured dictionary learning (IDSDL).
**Input:****N**_*t*_0_ −1_^*i*^, 
${\text{p}}_{s^{+}}^{i}$, 
${\text{p}}_{s^{-}}^{i}$, *i* = 1, 2, …, *N*, **T**_*t*_0_ −1_, *λ*_1_, *λ*_2_.1:
${\text{A}}_{0}^{+}\leftarrow 0$, 
${\text{B}}_{0}^{+}\leftarrow 0$, 
${\text{A}}_{0}^{-}\leftarrow 0$, 
${\text{B}}_{0}^{-}\leftarrow 0$2:**for**
*t* = *t*_0_→*T*
**do**3: Obtain 
${\text{P}}_{\left(t-1\right)}^{i}$, 
${\text{N}}_{\left(t-1\right)}^{i}$ by 
${\text{D}}_{\left(t-1\right)}^{i}$ based on Equation (4).4: **for**
*i* = 1 → *N*
**do**5:  Sparse representation of positive sample patches 
${\text{p}}_{s^{+}}^{i}$ to obtain 
$\beta_{s^{+}}^{i}\in {\mathbb{R}}^{d^{2}\times s^{+}}$ by Equation (2) based on **T***_t_*_−1_.6:  Sparse representation of negative sample patches 
${\text{p}}_{s^{-}}^{i}$ to obtain 
$\beta_{s^{-}}^{i}\in {\mathbb{R}}^{d^{2}\times s^{-}}$ by Equation (2) based on 
${\text{N}}_{t-1}^{i}$.7:  Update 
${\text{A}}_{t}^{+}\leftarrow {\text{A}}_{t-1}^{+}+\beta_{s^{+}}^{i}{\left(\beta_{s^{+}}^{i}\right)}^{T}$, 
${\text{A}}_{t}^{-}\leftarrow {\text{A}}_{t-1}^{-}+\beta_{s^{-}}^{i}{\left(\beta_{s^{-}}^{i}\right)}^{T}$8:  Update 
${\text{B}}_{t}^{-}\leftarrow {\text{B}}_{t-1}^{-}+{\text{p}}_{s^{+}}^{i}{\left(\beta_{s^{+}}^{i}\right)}^{T}$, 
${\text{B}}_{t}\leftarrow {\text{B}}_{t-1}+{\text{p}}_{s^{-}}^{i}{\left(\beta_{s^{-}}^{i}\right)}^{T}$.9:  **for**
*j* = 1 → *M*
**do**10:   Update 
$\left[{\left({\text{P}}_{t}^{1:N}\right)}^{j}\right]$ by Equations (7) and (8) with 
${\text{D}}_{t-1}^{i}={\text{P}}_{t-1}^{i}$.11:   Update 
${\text{d}}_{t}^{j}$ by Equations (7) and (8) with 
${\text{D}}_{t-1}^{i}={\text{N}}_{t-1}^{i}$.12:  **end for**13:  Update the learned dictionary part, 
${\text{P}}_{t}^{i}$, based on Equation (6).14:  Update the learned dictionary part, **D***_t_*, based on Equation (4).15: **end for**16: Update the template set, **T***_t_*, based on Equation (5).17:**end for****Output:**Updated **T***_t_*, **D***_t_*.


## 3. A Tracking Framework Based on IDSDL and K-Combined Voting SVM Classification

### 3.1. The Principle of Online Visual Tracking Based on Bayesian Inference

An online visual tracking problem can be interpreted as a Bayesian recursive and sequential inference task in a Markov model with hidden state variables and is further divided into cascaded estimation of a dynamical model and observation model [3]. Suppose a set of target images **Y***_t_* = {y_1_, y_2_,…, y*_t_*} have been given till time *t*; the hidden state vector of the target, represented as **X***_t_*, can be estimated as follows,

(9) $$p\left({\text{X}}_{t}|{\text{Y}}_{t}\right)\propto p\left({\text{y}}_{t}|{\text{X}}_{t}\right)\int p\left({\text{X}}_{t}|{\text{X}}_{t-1}\right)p\left({\text{X}}_{t-1}|{\text{Y}}_{t-1}\right)d{\text{X}}_{t-1}$$

where *p*(**X***_t_*|**X***_t_*_−1_) refers to the dynamical model between the two consecutive states and *p*(**y***_t_*|**X***_t_*) denotes the observation model related to the likelihood estimation of **y***_t_* based on the state, **X***_t_*.

In the context of particle filtering, typically, a set of candidates ${\tilde{\text{X}}}_{t}^{v}$, *v* = 1, 2, …,*V* is drawn from an importance distribution *q*(**X***_t_*|**X**_1:_*_t_*_−1_, **y**_1:_*_t_*) and the weights of the samples could be updated as:

(10) $$w_{t}^{v}=w_{t-1}^{v}\frac{p\left({\text{y}}_{t}|{\tilde{\text{X}}}_{t}^{v}\right)p\left({\tilde{\text{X}}}_{t}^{v}|{\tilde{\text{X}}}_{t-1}\right)}{q\left({\tilde{\text{X}}}_{t}|{\tilde{\text{X}}}_{1:t-1},{\text{Y}}_{t}\right)}$$

To avoid degeneration, the samples would be re-sampled according to the their corresponding importance weights to generate a set of equally-weighted particles. In the case of the bootstrap filter, *q* (**X***_t_*|**X**_1:_*_t_*_−1_,**y**_1:_*_t_*) = *p* (**X***_t_*|**X**_1:_*_t_*_−1_), where the weights become the observation likelihood, *p*(y*_t_*|**X***_t_*). This approximation is widely used in online visual tracking, because of its simplicity and efficiency Therefore, suppose the target state information at time *t* − 1 is known, and there is no other prior knowledge; an optimal state estimation result ***X***˜*_t_* is computed based on the maximum *a posteriori* (MAP) estimation of observations over *V* candidates sampling at time *t* and mathematically described by:

(11) $${\tilde{\text{X}}}_{t}=\underset{{\text{X}}_{t}^{v}}{\arg\max}p\left({\text{y}}_{t}|{\text{X}}_{t}^{v}\right)p\left({\text{X}}_{t}^{v}|{\text{X}}_{t-1}\right)$$

Based on the basic principle described, the details of dynamical modeling and observation modeling in the proposed framework are further described.

### 3.2. Gaussian Affine Warping for Dynamical Modeling

Ideally, a dynamical model *p*(**X***_t_*|**X***_t_*_−1_) should be able to fully describe the variation of the target in detail, yet in most practical cases, this could be approximately parameterized. Typically, at time *t*, the geometric parametrization of the target region can be realized by an affine transformation as:

(12) $${{\text{p}}^{\prime }}_{t}={\text{G}}_{t}\cdot {\text{p}}_{t}+{\text{t}}_{t}$$

where **p***_t_* = (*x_t_*,*y_t_*) and **p**′ = (*x′_t_*, *y′_t_*) correspond to the 2D coordinate before and after the transform separately, ${\text{G}}_{t}=\left[\begin{array}{cc} \theta_{t} & s_{t}\\ \alpha_{t} & \phi_{t} \end{array}\right]$ is an 2 × 2 non-singular matrix, referring to the composition of rotation and non-isotropic scaling and **t***_t_* = (*x_t_*, *y_t_*)^T^ is the 2D translation vector. In a homogeneous coordinate system, Equation (12) can be equivalently expressed as:

(13) $$\left[\begin{array}{c} {{\text{p}}^{\prime }}_{t}\\ 1 \end{array}\right]=\left[\begin{array}{cc} {\text{G}}_{t} & {\text{t}}_{t}\\ 0 & 1 \end{array}\right]\cdot \left[\begin{array}{c} {\text{p}}_{t}\\ 1 \end{array}\right]$$

Based on the principle above, Ross *et al.* [3] propose a variant of the particle filter, called affine warping, where the state of the target can be described as a six-tuple set, **X***_t_* = {*x_t_*,*y_t_*, *θ_t_*, *s_t_*,*α_t_*, *φ_t_*}, whose elements respectively denote *x*, *y* translations, rotation angle, scale, aspect ratio and skew direction. The elements of **X***_t_* are independently modeled by a Gaussian distribution around the previous state, as follows,

(14) $$p\left({\text{X}}_{t}|{\text{X}}_{t-1}\right)=N\left({\text{X}}_{t};{\text{X}}_{t-1},\Psi_{0}\right)$$

where **Ψ**_0_ is a vector whose elements are the corresponding variances of the affine parameters.

### 3.3. K-Combined Voting SVM Classification of Sparse Coefficients for Observation Modeling

The support vector machine (SVM) is one of the most widely used classifiers in machine learning and pattern recognition application. It makes attempts to find a separating hyperplane that maximizes the margin between two classes. The margin is defined as the distance of the closest point to the hyperplane. Given a set of instance-label pairs {*β_k_*, *l_k_*}, *k* = 1,2,…,*s*, *β_k_* ∈ ℝ *^n^*,*l_k_* ∈ {−1,+1}, it solves the following unconstrained optimization problem with a different loss function *ξ*(**w***_k_*; *β_k_*, *l_k_*) as:

(15) $$J(\text{w})=\underset{{\text{w}}_{k}}{\min}\frac{1}{2}{\left({\text{w}}_{k}\right)}^{T}{\text{w}}_{k}+c\sum_{k=1}^{s}\xi ({\text{w}}_{k};\beta_{k},l_{k})$$

where **w** is termed a support vector, which are the training patterns closest to the separating hyperplane, and *c* refers to the regulation term. In this paper, we apply the linear SVM version proposed by Fan *et al.* [31] for training and classification.

**Figure 4.**
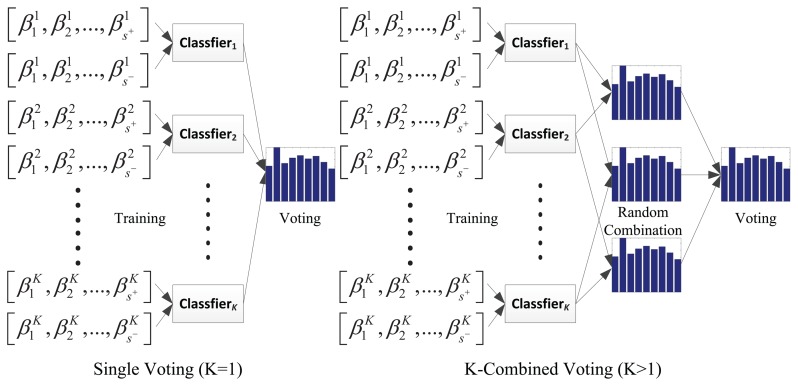
Single voting and random combined voting. The former scheme is a special case of the latter one with *K* = *1.*

We consider the discriminative observation modeling on a patch level. Given the patch-based coefficients, *β*, of candidate **X***_t_* are obtained and *P* candidates have been sampled, for the *p*-th candidate, *p* = 1, 2,…, *P*, a function called K-combined voting (KCV) is proposed to compute the score, *S*(p*), recording the times that p* is selected as the result by:

(16) $$p\left({\text{y}}_{t}|{\text{X}}_{t}\right)=\underset{p*}{\arg\max}\sum_{i=1}^{c_{N}}S(p_{i}^{*})$$

(17) $$S(p_{i}^{*})=1$$

(18) $$p_{i}^{*}=\underset{p}{\max}\left(\frac{1-\alpha }{1+e^{-{\text{w}}_{t-1}^{i}\beta_{p}^{C(i)}}}+\frac{\alpha }{1+e^{-{\text{w}}_{0}^{i}\beta_{p}^{C(i)}}}\right)$$

where C(i)=(NK)i, *i* = 1,2,…,*C_N_* refers to the *i*-th combinatory candidate given *K* > 1. When *K* = 1, it refers to the common single patch voting case, shown in Figure 4. ${\text{w}}_{0}^{i}$ refers to the support vector initially obtained for the *i*-th patch, while ${\text{w}}_{t-1}^{i}$ is vector generated at time *t* − 1. The K-combined voting can be seen as an efficient hierarchical generalization form of single voting. Based on each combination as the intermediate output, the final result provides a more comprehensive and neutral value within the sampled particles. In the case of drastic appearance variation, the random combined voting could provide more opportunities for the invariant patches to attend the voting calculation, so that it is more likely to obtain better results. Comparison between single voting and K-combined voting has been done as the proof in the next section. ${\text{w}}_{0}^{i}$, ${\text{w}}_{t-1}^{i}$ are weight vectors of the *i*-th classifier learned at the first frame and time *t* − 1, and *α* is a constant.

To both timely adapt the variation of target appearance and maintain its original invariance, a progressive classification is applied. At time *t*_0_, the voting function is processed twice, when sequentially, **w***_t_*_−1_ = **w***_t_*_0−1_ and **w***_t_*_−1_ = w_0_ are separately set. The former one is introduced to locate the target as an intermediate result based on its latest appearance model, while the latter one is used to locally refine the location with respect to its originality. Correspondingly, the dynamical modeling is also conducted twice to formulate a step-wise classification, similar to [15]. Thus, according to Equation (16), only the candidate most voted for is chosen as the estimation result.

### 3.4. Model Update

Once the current target location is estimated, the model is updated accordingly. In this paper, the update process is two fold. The first one is to adapt the dictionary using the proposed IDSDL algorithm proposed in the last section. Then, the positive and negative samples are sampled around the current estimated location of the target. Based on the learned results, sparse coefficients are obtained to train the SVM classifiers so that updated models are generated. Details of the IDSDL algorithm could refer to the last section, while the classifier training is described as follows.

To establish an efficient discriminative model at time *t* − 1, a local linear support vector classifier group ${\left\{{\text{Model}}_{t-1}^{i}\right\}}_{i=1}^{N}$ corresponding to each patch, is separately trained [31]. ${\left\{{\text{Model}}_{t-1}^{i}\right\}}_{i=1}^{N}$ contains the output support vectors, **w***_t_*_−1_, for current patch. For each ${\text{Model}}_{t-1}^{i}$, an object function, *J*(**w**), is established. The training data is generated based on the samples ${\text{Y}}_{t-1}=\left[{\text{p}}_{t-1}^{1},{\text{p}}_{t-1}^{2},\ldots ,{\text{p}}_{t-1}^{N}\right]$ drawn around the estimated target location and follow the same patch cropping pattern with that of target representation. For the *i*-th patch, the training data is made up of sparse coefficients by solving Equation (2) with ${\text{D}}_{t-1}^{i}$ as the dictionary. ${\text{p}}_{t-1}^{i}={\left\{\beta_{k}^{i},l_{k}^{i}\right\}}_{k=1}^{s}$, $\beta_{k}^{i}\in {\mathbb{R}}^{M\times N+M+2d^{2}}$, $l_{k}^{i}\in \left\{+1,-1\right\}$, separately correspond to the positive patches and negative ones of the candidates, and the *i–*th classifier for ${\text{Model}}_{t-1}^{i}$ is learned to minimize the loss function:

(19) $$J({\text{w}}_{t}^{i})=\underset{{\text{w}}_{k}^{i}}{\min}\frac{1}{2}{\left({\text{w}}_{k}^{i}\right)}^{T}({\text{w}}_{k}^{i})+c{\sum_{k=1}^{s}\left(\max\left(0,1-l_{k}^{i}{\left({\text{w}}_{k}^{i}\right)}^{T}\beta_{k}^{i}\right)\right)}^{2}$$

Totally, *N* classifiers are to be trained at each frame.

### 3.5. Summary of the Proposed Algorithm

The proposed algorithm is summarized in Algorithm 2 based on the descriptions above.



**Algorithm 2** Visual tracking based on IDSDL and K-combined voting SVM classification.
**Input**:Image sequence with *T* frames, initial target state **X**_0_, target region **Y**_0_, particle numbers *v*, overlapped percentage, *c_l_*, *c_s_*, *K, M*, *λ*_1_, *λ*_2_, **Ψ**_0_, *C* and *N*.**Output**:Current target state **X***_t_*1:*(Initialization)* Track the target during the first *M* frames to obtain the state, **X**_1:_*_M_*, and template set **T***_M_*.2:**for**
*t* = *M* + 1 → *T*
**do**3: *(Dynamical Modeling)* Obtain *V* target candidates 
${\left\{{\tilde{\text{y}}}_{t}^{v}\right\}}_{v=1}^{V}$ based on affine warping by Equation (14) with **Ψ_0_**.4: *(Observation Modeling)* Obtain the sparse coefficients of the candidates based on Equation (2).5: *(Observation Modeling)* Estimate the intermediate location of the target based on the multiple-linear-classifiers group by Equation (18) when **w***_t_* = **w***_t_*_o_.6: *(Dynamical Modeling)* Obtain *V* target candidates 
${\left\{{\tilde{y}}_{t}^{v}\right\}}_{v=1}^{V}$ based on affine warping by Equation (14) with **Ψ_0_**.7: *(Observation Modeling)* Obtain the sparse coefficients of the candidates based on Equation (2).8: *(Observation Modeling)* Estimate the location of the target based on the multiple-linear-classifiers group by Equation (18) when **w***_t_* = **w**_0_.9: *(Model update)* Sample the positive and negative samples around the current estimated location of the target.10: *(Model update)* Update **T***_t_*, **D***_t_* based on IDSDL by Algorithm 1.11: *(Model update)* Update Model*_t_* of multiple-linear-classifiers group based on Equation (19).12:**end for**


In Algorithm 2, the proposed dictionary learning algorithm is the most computational, while the online training and classification process does not take much running time, since the efficient linear SVM is applied. The dynamical modeling process takes the least running time according to the proposed straightforward process. To accelerate the process, we apply a C implementation of elastic net regulation proposed by Mairal *et al.* [30]. We also normalize the target patch to make it more efficient for data processing.

## 4. Experiment and Discussion

In this section, we present experiments on test videos to demonstrate the efficiency and effectiveness of the proposed algorithm.

### 4.1. Experiment Setup

The proposed tracking algorithm, IDSDL-VT, is implemented in MATLAB and C/C++ and runs at about 1.3 fps on a 3.4 GHz dual core PC with 8 GB of RAM. For parameter configuration, in each frame, each target region is normalized to 24 × 24, and the patch size is 12 × 12, *d* = 12, while the overlapped percentage of the neighbored patch is 0.5. Thus, *N* = 9. The number of particles is *V* = 600 for dynamical modeling. Moreover, the regularization constant, *λ*_1_ and *λ*_2_, in Equation (2) are set to 0.01, and the dictionary learning is processed once per frame. In Equation (18), *K* = 3, *α* = 0.5. During training, regions within two pixels around the target location are set as positive, while the ones in the outer four pixels are negative, and *c* = 10. Except Section 4.5, the target locations are manually labeled in the first five frames to generate the templates, *M* = 5.

To evaluate the efficiency of the proposed algorithms, nine benchmark video sequences, most of which are publicly available, are used under the challenges of lighting and scale changes, out-of-plane rotation and partial occlusion. Comparatively, the proposed tracker is evaluated against state-of-the-art algorithms, including Frag [4], IVT [3], VTD [16], L1T [11], TLD [10], MIL [9] and PLS [18]. The implementation is based on the source codes provided by the authors via their websites. Qualitative and quantitative evaluations are presented in the rest of this section.

It should be noted that the setting on a particle number and the regulation constant above is based on the setup of classical online visual tracking algorithms, for a better performance comparison [3,9–11,16]. Enlarging the normalized size of the target region and patch size would increase the computation time. Its current setting is established after the times of the experiments with reference to the related works. Moreover, the overlapped percentage of the neighbored patch is related to the appearance variation of the target region. Since a low percentage number would lead to lower efficiency and the benchmark video is of various kinds, a unbiased number, 0.5, is set. Values of *K* and *α* are empirically set based on the times of the experiments.

### 4.2. Qualitative Evaluation

Qualitative analysis and discussions are provided as follows. The visual challenges include heavy occlusion, illumination change, scale change, fast motion, cluttered background, pose variation, motion blur and low contrast.

**Figure 5.**
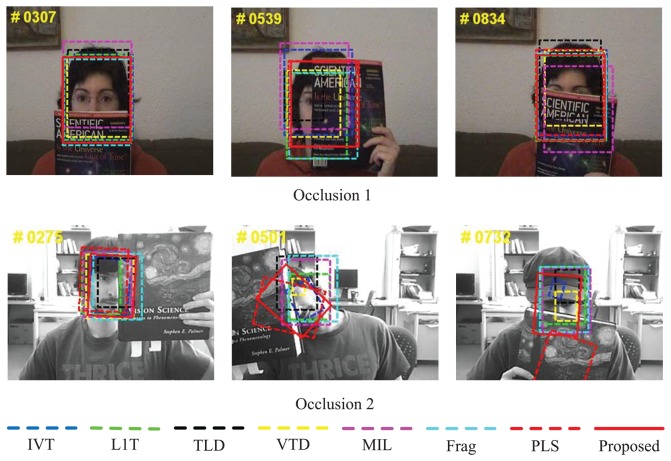
Qualitative evaluation results of eight algorithms on challenging tested sequences *Occlusion 1*and *Occlusion 2*.

The two test sequences, *Occlusion 1* and *Occlusion 2*, in Figure 5 are separate from the work by Adam *et al.* [4] and the one by Ross *et al.* [9], both of which highlight partial occlusion, and set the region of high-resolution human faces as the targets for tracking, which is widely used in human-computer-interface application environments. The frame numbers of the sequences are 898 and 819 of a size of 320 × 240. *Occlusion 2* is more challenging, because it also contains in-plane rotation and out-of-plane rotation. It is shown that for *Occlusion 1*, all the evaluation algorithms can follow the target approximately correctly, yet some of the algorithms deviate from the face when occlusion occurs (e.g., MIL [9] at #0307, #0539 and #0834, IVT [3], L1T [11], Frg [4], TLD [10] and VTD [16] at #0539). For *Occlusion 2*, the differences are more obvious. It can be found that L1T [11] drifts more from the target compared with other algorithms (e.g., MIL [9] at #0501 and #0732), and IVT [3] and TLD [10] cannot adapt the appearance when there are occlusion and head rotation (e.g., #0732). PLS [18] cannot continuously follow the target, while MIL [9] and Frag [4] estimate the target less accurately than the proposed algorithm.

**Figure 6.**
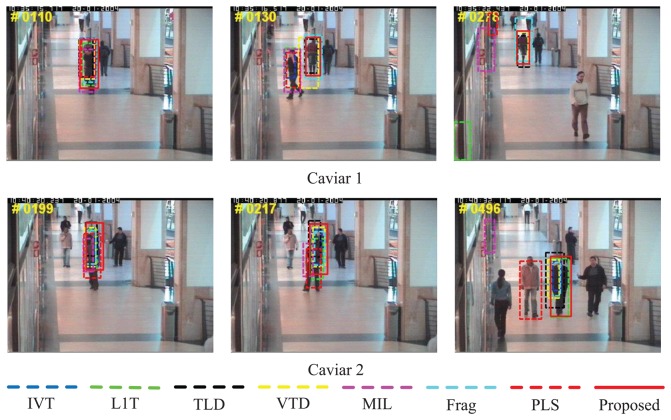
Qualitative evaluation results of eight algorithms on challenging tested sequences *Caviar 1* and *Caviar 2*.

The sequences, *Caviar 1* and *Caviar 2*, in Figure 6 come from the CAVIAR project (http://groups.inf.ed.ac.uk/vision/CAVIAR/) with the frame numbers 382 and 500 of a size of 384×288. Both of them comprise severe partial occlusion and scale variation from far to near, which are typical scenes in surveillance applications. Moreover, there are similar objects near the target as the distractions. It is be shown that MIL [9], L1T [11] and PLS [18] do not perform well in *Caviar 1*. The first two methods fail to discover the target when the target is occluded by a similar object (e.g., #0130), while the latter one drifts away from the target (e.g., #0130 and #0218). Only the proposed tracker, VTD [16], Frag [4] and TLD [10], handle the heavy occlusion successfully. However, Frag [4] cannot smoothly adapt the scale changes of the person (e.g., #0278). In *Caviar 2*, almost all the trackers evaluated, except PLS [18] and MIL [9], can follow the target. However, many of them, including IVT [3], VTD [16] and TLD [10], cannot adapt the scale as the human moves near the camera (e.g., #0217 and #0496). In contrast, our algorithm performs well in terms of position estimation and scale adaptation.

**Figure 7.**
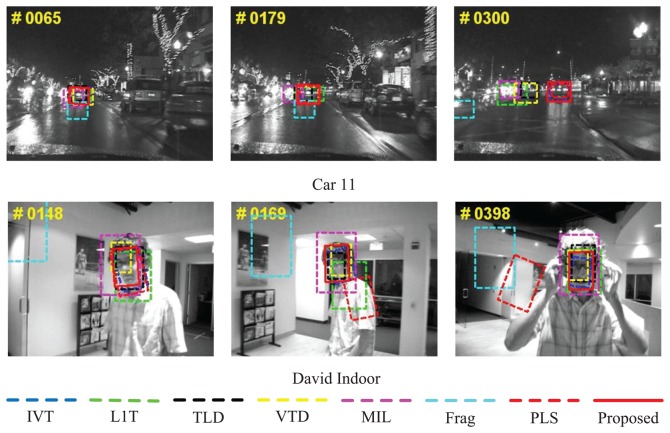
Qualitative evaluation results of eight algorithms on challenging tested sequences *Car 11*and *David Indoor*.

The sequences, *Car 11*and *David Indoor*, in Figure 7 are from the work by Ross *et al.* [3] with the frame numbers 659 and 462 of a size of 720 × 480 and 320 × 240. *Car 11*are quite common in practical intelligent vehicle application environments and are very challenging, as this is a video at night. The target (the rear view of a car) is small and easily distracted by the surroundings, including similar vehicle appearance and glare. It is shown that only IVT [3], PLS [18] and the proposed algorithm successfully can track the target in the whole sequence, while the remaining drift away or take the surroundings as the target (e.g., MIL [9] at #0065, #0179 and #0300 and VTD [16] and L1T [11] at #0179 and #0300). *David Indoor*contains out-of-plane rotation as the person turns his or her face and scale change, due to distance variation from the cameras. It also contains illumination changes, as the person walks from a dark room into areas with a spot light. For this sequence, some algorithms (e.g., Frag [4] and PLS [18]) drift away from the target during the tracking process, while some algorithms can not adapt the scale when out-of-plane rotation occurs (e.g., MIL [9] and L1T [11] at #0169 and #0398). Comprehensively and qualitatively speaking, the proposed algorithms perform the best.

**Figure 8.**
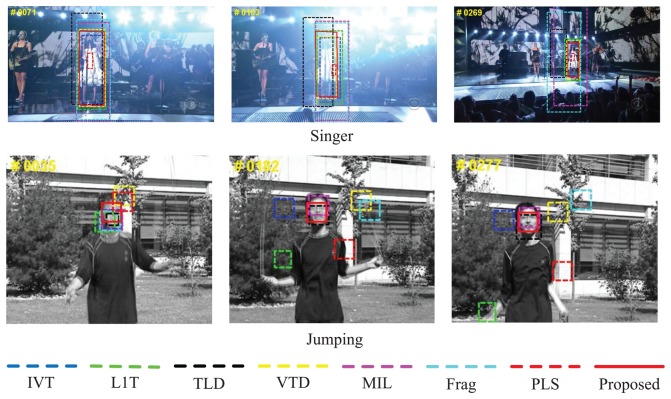
Qualitative evaluation results of eight algorithms on challenging tested sequences *Singer*and *Jumping*.

The two video sequences, *Singer*and *Jumping*, in Figure 8 are from the work by Kwon *et al.* [16] and TLD [10]. The frame numbers of the sequences are 321 of a size of 624 × 352 and 313 of a size of 352 × 288. *Singer*is challenging, as it contain illumination variation, and *Deer*highlights abrupt motions. In the former one, only the results of partial trackers (e.g., the proposed algorithm and VTD [16]) are satisfactory, while the others cannot adjust the scale (e.g., Frag [4], L1T [11] and MIL [9]) or accurately locate the target (e.g., TLD [10] at #0071, #0103 and #0269; IVT [3] at #0126) a drastic scale and location deviation appears when lighting conditions change. Especially PLS [18] cannot capture the scale variation of the target through all the frames of *Singer*. In *Jumping*, the successful trackers only include the proposed algorithms, MIL [9] and TLD [10], while the others fail to capture the head of the person when he or she jumps up and down repeatedly. Comprehensively and qualitatively speaking, the proposed algorithms perform the best.

### 4.3. Quantitative Evaluation

Besides qualitative evaluation, quantitative evaluation of the tracking results is also an important issue for tracking performance evaluation. Similar to other classical works, two performance measurements are applied to compare the proposed tracker with the other reference trackers. Quantitative comparisons using average center errors (CE) based on Euclidean distance and the PASCAL [32] overlap rate (OR) criterion between the proposed method and the other ones are conducted.

Moreover, the average center error (ACE) and average overlap rate (AOR) are defined as:

(20) $$\text{ACE}=\frac{1}{T}\sum_{i=1}^{T}{\left\|{\text{c}}_{\text{eval}}^{i}-{\text{c}}_{gt}^{i}\right\|}_{2}^{2}$$

(21) $$\text{AOR}=\frac{1}{T}\sum_{i=1}^{T}\frac{{\text{A}}_{\text{eval}}^{i}\cap {\text{A}}_{gt}^{i}}{{\text{A}}_{\text{eval}}^{i}\cup {\text{A}}_{gt}^{i}}$$

where ${\text{c}}_{\text{eval}}^{i}$, ${\text{c}}_{gt}^{i}\in {\mathbb{R}}^{2\times 1}$ refer to the horizontal and vertical center coordinates of the evaluation and ground-truth labeling results at the *i*-th frame, respectively, and ${\text{A}}_{\text{eval}}^{i}$, ${\text{A}}_{gt}^{i}\in {\mathbb{R}}^{+}$ are corresponding areas of the target in one test sequence. *T* refers to the frame number of the test sequence. The results for each sequence and each method are shown in Table 1, and it can be concluded that the proposed tracking method performs more favorably than the other methods and the single-voting case. Though some CE values are higher, the gaps are significantly limited, and both OR values and two criterion averages on all tested sequences of the proposed tracker are better than all the other ones.

To demonstrate the proposed improvement in the voting scheme, comparison between single voting and K-combined voting is also drawn on the benchmark sequences. The settings are the same with the ones above. It can be found that the proposed algorithm with K-combined voting is better in both center error evaluation and overlap rate evaluation. Even with single voting, the proposed tracker can perform better than other classical trackers on the overlap rate in most cases. It should be noted that, on the one hand, the performance with a low *K* would approach that in the single voting case. On the other hand, it could introduce the classification error when *K* is too high. In our experiments, we find that the trackers perform very well when *K* = 3.

**Figure 9.**
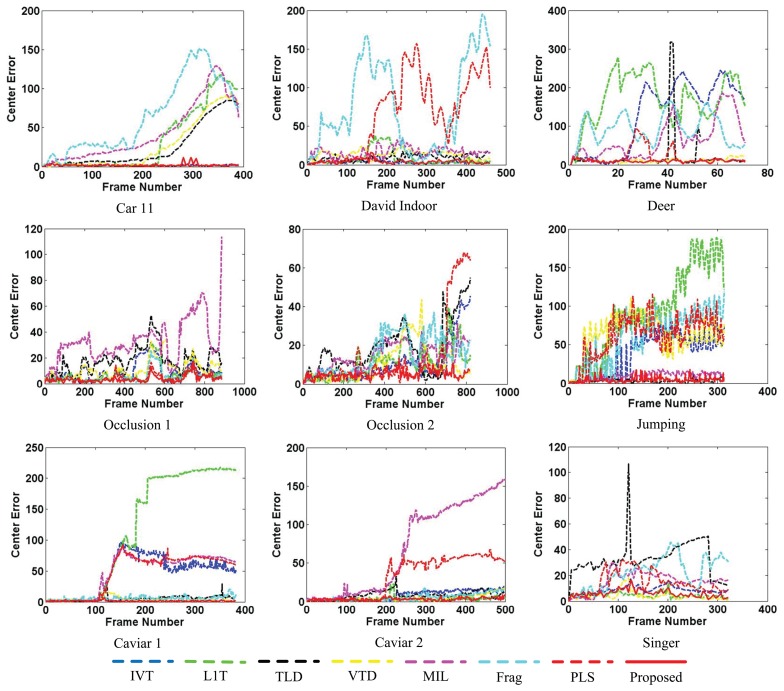
Center error (CE) evaluation for nine video clips. The proposed algorithm is compared with seven state-of-the-art methods: Frag[4], IVT [3], VTD [16], L1T [11], MIL [9], TLD [10] and PLS [18].

**Figure 10.**
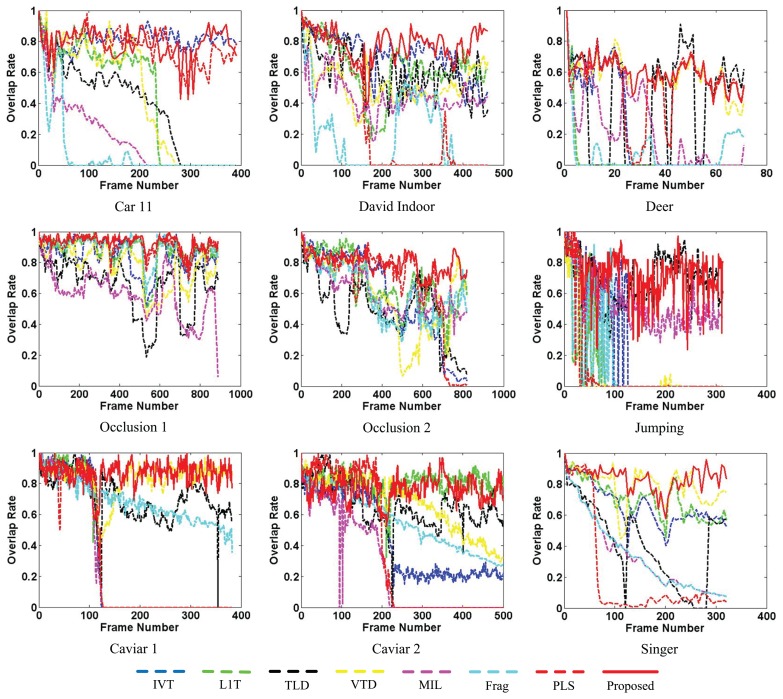
Overlap rate (OR) evaluation for nine video clips. The proposed algorithm is compared with seven state-of-the-art methods: Frag [4], IVT [3], VTD [16], L1T [11], MIL [9], TLD [10] and PLS [18].

Figures 9 and 10 separately illustrate the center error and overlap rate figures for all the quantitatively evaluated sequences. Based on these figures, it can be seen that our proposed algorithm can obtain narrow ranges of fluctuations against the other algorithms (e.g., *David Indoor* and *Jumping*). Though the values of the proposed tracker are not the best all the time, they are lower in the center error and higher in the overlap rate than the other algorithms in most test frames. Thus, the proposed tracker provides comprehensively more favorable results in CEE and AOR averages than the other algorithms described in Table 1.

Overall, it can be concluded that the proposed tracker achieves better performance than the other state-of-the-art algorithms.

### 4.4. Dictionary Learning Results and Time

**Figure 11.**
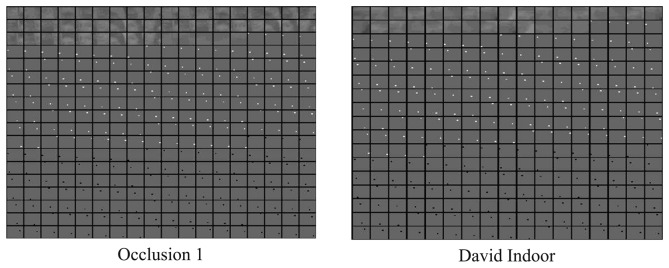
Dictionary of *Occlusion 1* and *David Indoor* learned by IDSDL after 100 frames (best viewed in color).

In this paper, a dictionary learning algorithm called incremental discriminative structured dictionary learning (IDSDL) is proposed to learn from positive and negative samples, joined to construct a structured dictionary with a newly established randomly permuted unit matrix for sparse representation. Each test sequence corresponds to a dictionary during the tracking process. Corresponding to the patch settings above, the selected learned dictionaries of sequence *Occlusion 1* and *David Indoor* after 100 frames are shown in Figure 11 to demonstrate the proposed dictionary design and learning results. The values have been normalized before plotting for better illustration.

Moreover, the average computation time per frame of the proposed IDSDL algorithm based on different target normalized sizes are provided in Table 2. We take the challenging sequence, *Car 11* as an example. The corresponding patch size is a quarter of the whole, and other parameter settings are the same as those described in the beginning of this section. The corresponding ACE and ORE are also provided. It can be shown that as the normalized size decreases, the running time gets shorter, yet the ACE and ORE get worse correspondingly. The proposed tracker fails to continuously track the target when the target patch is normalized to 8 × 8. This is because the details of the target are lost when the target region is interpolated on a more coarse-grained scale, and thus, the discrimination ability could not be satisfactorily maintained. In our experiments, the normalized size is established after the times of the experiments on all the test sequences with reference to the balance between accuracy and efficiency.

### 4.5. Extending to Relay Tracking in Visual Sensor Networks

**Figure 12.**
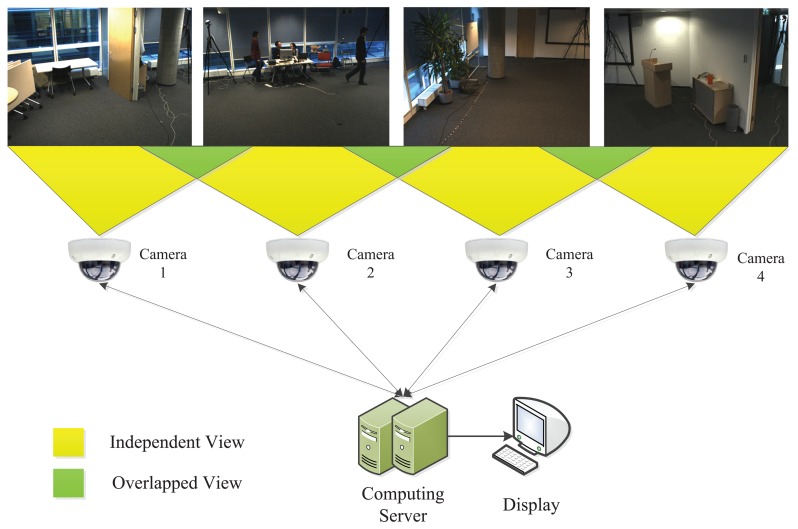
Relay tracking evaluation establishment. We assume that the cameras are connected by the local area network (LAN) with the computing server in the back-end. The videos acquired would be transmitted to the server without any time delay.

To demonstrate the potential application of the proposed algorithm, we evaluate its relay tracking performance in visual sensor networks. The test dataset is from the CATproject (http://www. cat-project.at/). There are four cameras, and their fields of view are slightly overlapping. A person walks across the four cameras with different view points, which is regarded as the target. Our evaluation is established as shown in Figure 12. We assume that the cameras are connected by the local area network (LAN) with the computing server in the back-end. The videos acquired would be transmitted to the server without any time delay. Moreover, each camera corresponds to a tracker in the server.

**Figure 13.**
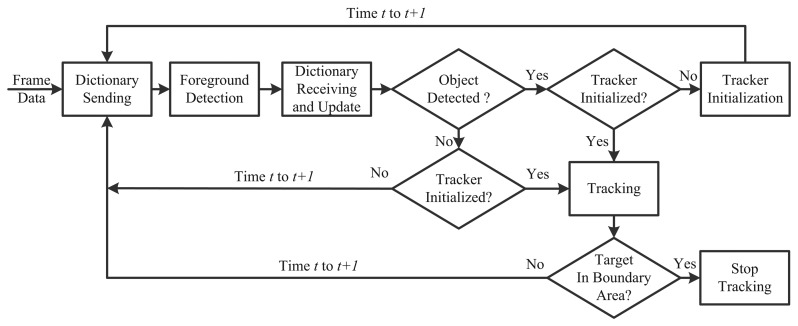
Tracking process with a shared dictionary across all the cameras.

In order to make use of the visual information acquired as much as possible, we establish the tracking process with a shared dictionary across all the cameras, shown in Figure 13. When the cameras are switched on, their trackers begin to work. Here, we assume that the person entering the scene is the one we are going to track. We apply foreground extraction based on Gaussian background modeling [33] to detect the newly appeared person in the boundary area (5% of the frame height and width in this paper). When the foreground area is larger than a predefined threshold, the person is considered to be detected. Once one camera detects the target, it records the corresponding location and starts to track the target. If there is no foreground detected, the process sends the dictionary learned during the tracking process in this camera. All the other trackers corresponding to different cameras would replace the old dictionary with a newly received one, so that the visual information on the dictionary level can be shared across the network. An empty dictionary is also sent in the no foreground detection and no tracking case. For a straight forward implementation, the person entering the boundary area for the second time is considered as a disappearance, so that the tracking process in the current camera stops.

Quantitatively, we evaluate the lifecycle of the target once it is detected in one camera. In this paper, the lifecycle of a target is defined as:

(22) $$p_{lc}=\frac{N_{c}}{N_{e}}$$

where *N_c_* is the frame numbers where the target is correctly tracked, and *N_e_* is the frame numbers where the target actually appears in the scene. In most cases, *N_e_* > *N_c_*. When the target is tracked continually once it enters the scene, *N_e_* = *N_c_*. The target is regarded as being correctly followed when OR is higher than 0.5. We compare the lifecycle of the Kalman tracking algorithm and the proposed algorithm without and with dictionary share. The result of the lifecycle is shown in Table 3.

Vertically, it can be found from the table that the proposed algorithm with dictionary sharing achieves higher values. This is mainly because of the satisfactory online tracking performance proposed above. Moreover, based on dictionary sharing, more visual information about the target could be obtained before the target enters the specific scene. Thus, the performance with the dictionary sharing is better than the one without sharing. Horizontally, all the values in Camera 2 and Camera 4 are lower than their counterparts in other columns. This is due to the background modeling in Camera 2 and Camera 4. In Camera 2, there are other moving objects as the target enters the scene, and in Camera 4, there is light variation. The foreground target could not be timely and correctly detected, which leads to a relatively poor lifecycle performance. Moreover, the value with the dictionary sharing in Camera 2 is not much higher than that without sharing, yet the opposite case occurs in Camera 3. This is because, due to the camera view point, the person's initial pose in Camera 2 is much different from those in other cameras. Thus, the corresponding dictionaries learned in other cameras could not provide much effective information about the target. It should be noted that the Kalman tracking method heavily and continuously relies on the background modeling performance. It could not track the target until its foreground is re-detected again, and due to the variation of foreground area, the target is not correctly labeled in some frames. Comparatively, our proposed tracker only relies on the foreground information once for location initialization, and with the dictionary sharing across the network, it achieves a better performance.

### 4.6. Discussion

**Figure 14.**
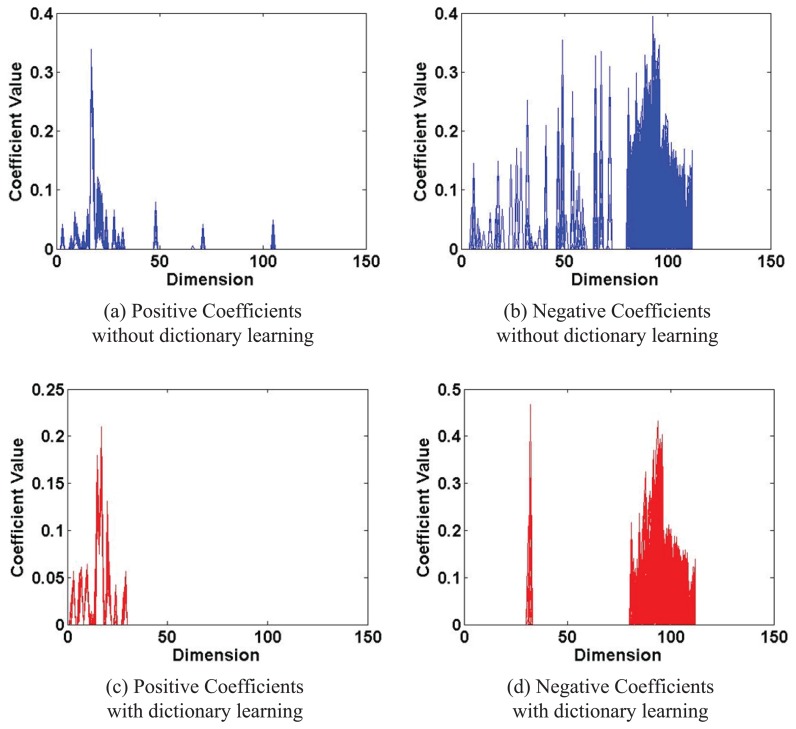
Coefficients of positive and negative samples in sequence *Car 4*with/without dictionary learning. The parameter settings are the same with those in the experiments above.

It can be found that our proposed tracker could perform more favorably than the other state-of-the-art trackers comprehensively in both qualitative and quantitative evaluation. We present some justifications here. For discriminative tracking algorithms, discrimination of the target from the background is critical. One of our contributions is the proposed IDSDL algorithm for dictionary learning. The proposed dictionary contains both a positive template set and negative samples, and during training, the LU strategy is proposed to only update its partial columns. Furthermore, the positive samples are used to update the positive part, and their negative counterparts are used to generate the learned negative part. Figure 14 shows the coefficients of positive and negative samples at #0016 in sequence *Car 4*with/without dictionary learning. These coefficients would be the input of the SVM classifiers for training. It can be found that, without dictionary learning, the coefficient values are more globally distributed across the dimension, while with dictionary learning, the data are more aggregated. Therefore, the proposed dictionary learning algorithm can improve the discrimination ability of the sparse coefficients, so that better classifiers and tracking performance could be obtained.

Confidence is also important for observation estimation. Our proposed KCV voting method combines the classifiers randomly and outputs their estimated result by a maximal scheme. Since the candidates are also generated randomly, the random combination could also be viewed as a supplementary re-sampling step from the particle filtering aspect. A limited combination of random sample points are still random, because the joint distribution of single Gaussian variables is still Gaussian. Thus, statistically speaking, it improves the estimation generalization during the tracking process. The maximal scheme is a nonlinear superposition process, and it creates more confidence points given limited candidates.

It has been shown from the experiments that our proposed tracker is currently not sufficient for real-time processing. Currently, it is mainly a MATLAB implementation with some C/C++ Mex functions. Most of the processing time is spent on the dictionary learning and classifier training part. It is certain that the processing could be several times faster in both single camera and visual sensor network cases when all the codes are re-written in C. The running speed could be higher if the processing could be paralleled or assisted with a graphic processing unit (GPU) coprocessor, since each patch could be independently processed before KCV without interleaving. Though the proposed tracker is slower, it achieves better performance in the accuracy evaluation.

## 5. Conclusions

This paper proposes an online discriminative visual tracking algorithm based on incremental discriminative structured dictionary learning and multiple linear classification based on randomly-combined voting. Not only qualitative, but also quantitative, evaluations are conducted, which demonstrate that, on challenging image sequences, the proposed tracking algorithm enjoys better performance than the state-of-the-art algorithms. It is also shown that our proposed algorithm could be applied to relay tracking with satisfactory performance in visual sensor networks. Our future work might focus on the application of the proposed dictionary learning method to other classification problems. The proposed algorithm could also be extended to multiple object tracking or the tracking of specific class (e.g., humans or their parts) given certain application environments.

## Figures and Tables

**Table 1.** Center error (pixels) and overlap rate of the tracking methods. The best three results are in bold, italicized and underlined fonts.

	Frag	IVT	VTD	L1T		TLD	MIL	PLS	Proposed	Single Voting
Occlusion 1	5.621	9.175	11.135	6.500		17.648	32.260	***4.596***	**3.656**	3.921
	0.899	0.845	0.775	0.876		0.649	0.594	***0.904***	**0.932**	0.910
Occlusion 2	15.491	***10.212***	10.408	11.119		18.588	14.058	46.186	**4.501**	4.931
	0.604	0.588	0.592	***0.672***		0.493	0.612	0.471	**0.813**	0.753
Caviar 1	5.699	45.245	***3.909***	119.932		5.593	48.499	47.393	**1.662**	1.696
	0.682	0.277	0.834	0.278		0.704	0.255	0.268	**0.868**	***0.813***
Caviar 2	5.569	8.641	4.724	3.243		8.514	70.269	32.431	**3.238**	***3.252***
	0.557	0.452	0.671	0.811		0.658	0.255	0.365	**0.814**	***0.802***
Deer	92.089	127.467	***11.920***	171.468		25.652	66.457	20.198	**9.763**	9.833
	0.076	0.217	0.577	0.039		0.412	0.213	0.510	**0.598**	***0.566***
Car 11	63.922	2.106	27.055	33.252		25.113	43.465	**1.691**	1.907	***2.052***
	0.086	***0.808***	0.432	0.435		0.376	0.175	0.769	**0.817**	0.811
David Indoor	76.691	**3.589**	13.552	7.630		9.671	16.146	64.335	4.177	***4.763***
	0.195	***0.712***	0.525		0.625	0.602	0.448	0.278	**0.783**	0.755
Singer	22.034	8.483	**4.057**	4.571		32.690	15.171	14.199	4.942	***5.426***
	0.341	0.662	***0.790***	0.703		0.413	0.337	0.212	**0.837**	0.809
Jumping	58.448	36.802	62.988	92.393		**3.589**	9.894	60.206	4.051	***4.471***
	0.138	0.283	0.080	0.093		0.690	0.527	0.096	**0.724**	***0.664***

**ACE** **Average**	38.396	27.969	16.639	50.012		***16.340***	35.135	32.359	**4.211**	4.483
**AOR** **Average**	0.398	0.538	***0.586***	0.504		0.555	0.380	0.430	**0.798**	0.765

**Table 2.** Running time, average center error and ORE of different normalized patch sizes for *Car 11*.

**Normalized Size (Pixels)**	**Running Time (Seconds)**	**ACE (Pixels)**	**AOR**
32 × 32	3.704s	1.826	0.821
24 × 24	1.192s	1.907	0.817
16 × 16	0.930s	4.022	0.783
8 × 8	0.433s	25.441	0.406

**Table 3.** The lifecycle of different methods. The best results are in bold font.

	**Camera 1**	**Camera 2**	**Camera 3**	**Camera 4**	**Average**
Kalman	0.624	0.607	0.705	0.501	0.609
Proposed (no dictionary share)	0.806	0.709	0.753	0.641	0.727
Proposed (dictionary share)	**0.873**	**0.732**	**0.884**	**0.702**	**0.798**
